# Long-term obstetric, perinatal, and surgical complications in singleton pregnancies following previous cesarean myomectomy: a retrospective multicentric study

**DOI:** 10.3389/fsurg.2024.1430439

**Published:** 2024-08-01

**Authors:** Oğuz Güler, Şafak Hatırnaz, Radmila Sparic, Alper Basbug, Onur Erol, Üzeyir Kalkan, Hasan Ulubaşoğlu, Giuseppe Trojano, Sebati Sinan Ürkmez, Andrea Tinelli

**Affiliations:** ^1^ Department of Obstetrics and Gynecology, Private Asya Hospital, Istanbul, Turkey; ^2^ Department of Obstetrics and Gynecology, Mediliv Medical Center, Samsun, Turkey; ^3^ Clinic for Obstetrics and Gynecology, University Clinical Center of Serbia, Belgrade, Serbia; ^4^ Faculty of Medicine, University of Belgrade, Belgrade, Serbia; ^5^ Department of Obstetrics and Gynecology, School of Medicine, Duzce University, Duzce, Turkey; ^6^ Department of Obstetrics and Gynecology, Memorial Antalya Hospital, Antalya, Turkey; ^7^ Department of Obstetrics and Gynecology, Koç University Hospital, Istanbul, Turkey; ^8^ Department of Obstetrics and Gynecology, Ankara Bilkent City Hospital, Ankara, Turkey; ^9^ Department of Obstetrics and Gynecology, Madonna Delle Grazie Hospital, Matera, Italy; ^10^ Department of Medical Biochemistry, Faculty of Medicine, Ondokuz Mayıs University, Samsun, Turkiye; ^11^ Department of Obstetrics and Gynecology, and CERICSAL (CEntro di RIcerca Clinico SALentino), “Veris delli Ponti Hospital”, Scorrano, Italy

**Keywords:** cesarean myomectomy, endometrial myomectomy, serosal myomectomy, uterine fibroids, myoma recurrence, pregnancy, adhesions, complications

## Abstract

**Objectives:**

The safety of cesarean myomectomy has been proven by previous studies. Our study aimed to reveal the long-term perinatal, obstetric, and surgical outcomes of cesarean myomectomy (CM) by comparing different CM techniques.

**Material and methods:**

This retrospective multicentric case–control study involved 7 hospitals and included 226 singleton pregnancies that underwent repeated cesarean section (CS) between 2015 and 2020. Among these pregnancies, 113 of 226 cases had CM (Group A), and 113 had only CS (Group B). Of the 113 cases in which CM was performed, 58 underwent endometrial myomectomy (EM) (Subgroup A1) and 55 underwent serosal myomectomy (SM) (Subgroup A2). The groups were compared in terms of obstetric, perinatal, and surgical outcomes, and fibroid recurrence, myomectomy scar healing rate, and adhesion formation were noted.

**Results:**

There was no significant difference between the groups in terms of maternal age, body mass index, gravidity, parity, and fibroid diameter in previous CS (*p* > 0.05). In the perinatal and obstetric evaluation of the groups, there was no significant difference between the groups in terms of neonatal weight, Apgar score, fetal growth restriction, preterm premature rupture of membranes, preterm delivery, hypertension in pregnancy, and diabetes mellitus (*p* > 0.05). The fibroid recurrence rate was 28.3%, and the myomectomy scar good healing rate was 99.1%. There was no difference between the groups in terms of CS duration, preoperative and postoperative hemoglobin levels, perioperative blood transfusion rates, febrile morbidity, and prolonged hospitalization (*p* > 0.05). In terms of adhesion formation, although the adhesion rate of the SM group was higher than that of the EM group, no statistically significant difference was detected between the groups.

**Conclusion:**

This study showed that in pregnancies following CM, obstetrical, perinatal, and surgical outcomes were unaffected. Obstetricians can safely use CM, either the trans-endometrial or serosal technique, as it is a safe and effective method with long-term results.

## Introduction

The most common benign tumors in the female reproductive system are fibroids or myomas, which can pose a significant health risk, especially to expectant mothers (1). Since childbearing is becoming more and more of a worldwide trend, this load has grown in importance in recent decades (2). Although estimates of the prevalence of fibroids in pregnant women vary, a conservative estimate for those found during regular second-trimester ultrasounds is 2.7%. When a woman is having *in vitro* fertilization (IVF), this incidence rises to 12.6%, and it may even reach 25% in older women who are doing IVF with an ovum donor (3).

Most fibroids do not cause any major problems during pregnancy, delivery, or puerperium; however, they are linked to several perinatal issues, including miscarriage, early pregnancy bleeding, premature labor and rupture of membranes, placental abruption, malpresentation, labor dystocia, postpartum hemorrhage, and retained placenta (4).

Cesarean myomectomy (CM), a procedure involving myomectomy performed during cesarean delivery, was first reported in 1913. For an extended period, it was considered a hazardous operation due to the pronounced risk of massive perioperative hemorrhage, which often necessitated a hysterectomy to achieve hemostasis (5). However, since the last decades of the previous century, CM has gained increasing support, attributed to its numerous advantages over interval myomectomy (6). Despite initial reservations, five meta-analyses published in the past decade have not demonstrated significant complications associated with myomectomy during cesarean section (CS), compared to CS alone (7–10). Traditionally, CM is performed using a serosal uterine incision hysterotomy. Yet, recent reports have indicated that CM can be effectively conducted via an endometrial incision in suitable cases, yielding favorable outcomes (11).

There is a lack of data on the long-term outcomes of CM, with only a limited number of reports available in the existing literature (12, 13). Additionally, none of the meta-analyses published to date have included data on this aspect (7–10). The primary aim of this study was to investigate the perinatal and perioperative complications during pregnancy and delivery in women subjected to CM during previous delivery. The secondary aim was to determine if there is a difference in the frequency of these complications according to the CM technique used.

## Material and methods

This study was designed as a retrospective, multicentric case–control investigation conducted across seven hospitals, comprising four tertiary-level and three secondary-level facilities. The study population consisted of women who underwent repeated CS in singleton pregnancies over a 5-year period from 2015 to 2020. Ethics committee approval was obtained for all the participating hospitals, and the study was registered at clinicaltrials.gov, with No. NCT04766567. Informed consent was obtained and signed by all the patients included in the study. The enrolled women were categorized into two groups: (1) Group A comprised patients who have had CM during previous CS. The inclusion criteria for CM included patients expressing a desire for the procedure after being informed about its risks and benefits before delivery. The exclusion criteria for CM included patient refusal, uterine hypotony, and/or atony, cervical myomas that cannot be reached from the abdomen, and women with bleeding disorders due to medical or obstetric causes. (2) Group B comprised patients who have had CS only. Due to the large number of potential participants in Group B, patients who matched Group A in terms of age and BMI were identified. Subsequently, an equal number of patients to Group A were selected from this pool, using an online random picker tool. Then, further subdivision of Group A was conducted into the following: (3) Subgroup A1, all patients who underwent endometrial myomectomy (EM), and (4) Subgroup A2, all women who underwent serosal myomectomy (SM). We excluded women who have had any other intra-abdominal surgery except CS (with or without CM), those having more than one previous CS, those having any coagulopathy, and cases of repeated CM. Patients with a history of malignancy, pelvic inflammatory disease, and endometriosis were also excluded from the study. No anti-adhesion materials were used during previous CS and CM of the cases. All CSs were performed according to the technique previously described by Stark (14). SMs were performed in all the cases according to the technique previously described by Tinelli et al. (15). EMs were performed in all the cases according to the technique previously described by Hatirnaz et al. (11). The medical charts were reviewed, and the following sociodemographic and clinical data were extracted: age at repeated CS, gravidity and parity, gestational age at delivery, time interval since previous CS, type of previous laparotomy, neonatal weight, and Apgar score at 1 and 5 min. The following pregnancy complications were registered for all the participants: fetal growth restriction (FGR), intrauterine fetal demise, diabetes mellitus (DM), hypertensive disorder in pregnancy, preterm premature rupture of membranes (PPROM), preterm delivery, placenta previa, placental abruption, abnormally invasive placenta (AIP), uterine rupture, and venous thromboembolism. Perioperative characteristics were also recorded for all the patients, including the kind of relaparotomy used for the repeated CS, duration of the CS (from skin incision to skin closure in minutes), preoperative and postoperative serum hemoglobin levels, transfusion frequency, postoperative intestinal sub-occlusion/occlusion, febrile morbidity, and prolonged hospitalization (≥2 days longer than the institutional average). Postoperative serum hemoglobin levels were measured 12 h after the CS. Febrile morbidity was defined as temperature ≥38 °C measured at least twice at an interval of at least 6 h between the two measurements, except within the first postoperative 24 h. Healing of the previous CM scar in Group A was evaluated by intraoperative inspection and with palpation, and the findings were classified into three categories, namely, good healing (scar thicker than 50% of the surrounding uterine wall), poor healing (scar thinner than 50% of the surrounding uterine wall), and scar dehiscence. The recurrence rate of fibroids in Group A was determined from the presence of fibroids during the repeated CS. In cases of fibroid presence, the diameter of fibroids was registered from the operative records. Surgical complications associated with the repeated CS were evaluated according to Clavien–Dindo classification (16), in all cases, as absent, Grade I (any deviation from the normal postoperative course: drugs as antiemetics, antipyretics, analgesics, diuretics, electrolytes, physiotherapy, and wound infections opened at the bedside), Grade II (drugs other than such allowed for Grade I complications, blood transfusions, and total parenteral nutrition), and Grade III and above.

The presence of postoperative adhesions registered during repeated CS was evaluated according to the peritoneal adhesion index (PAI) (17). In the PAI system, the human abdomen is divided into nine equal anatomical regions. The grade score of peritoneal adhesions in these regions is defined as 0, no adhesions; 1, filmy adhesions; 2, strong adhesions; and 3, very strong vascularized adhesions. The sum of the adhesion grade scores of nine regions results in PAI.

The data in the study were analyzed using IBM SPSS Statistics for Windows v 21.0 (IBM Corp, Armonk, NY, USA). The quantitative data are presented as the median (minimum–maximum) values. The categorical data are presented as frequencies (*n*) and percentages (%). Kruskal–Wallis's test and Mann–Whitney *U* test were used to compare the independent groups, and Pearson's chi-square test, Fisher's exact test, and Fisher–Freeman–Halton test were used to compare the categorical variables. Logistic regression analyses were conducted to calculate the odds ratios to explore the relationship between surgical techniques and both long-term postoperative complications and the incidence of intraoperative adhesions. The regression models were constructed in two phases for the entire study population. Initially, univariate models were developed. Subsequently, multivariate models were adjusted for age, BMI, gravidity, parity, and the time interval between operations. These analyses were replicated exclusively within the CS myomectomy group to assess the impact of myoma diameters in the initial CS/myomectomy operations. Initially, univariate models were established. This was followed by the development of multivariate models, which were adjusted for age, BMI, gravidity, parity, time interval between operations, and myoma diameter at the initial operations. Data were determined at the 95% confidence level, and a *p*-value of <0.05 was accepted as statistically significant.

## Results

A total of 226 women submitted for repeated CS were included in the study. Participants were divided into two main groups: Group A comprising 113 patients who have had either serosal myomectomy (SM) or endometrial myomectomy (EM) and Group B comprising 113 patients who have had CS only. Group A was further subdivided into two subgroups: Subgroup A1 consisting of 58 patients who have had EM and Subgroup A2 consisting of 55 patients who have had SM. Myomas enucleated in Subgroup A1 were G1, G2, G3, G4, G5, and G6 [by the International Federation of Gynecology and Obstetrics (FIGO) leiomyoma subclassification system] myomas. The myomas enucleated in Subgroup A2 were G4, G5, G6, and G7 myomas. The clinical characteristics of patients within the two primary groups (Group A and Group B), as well as the two subgroups (Subgroup A1 and Subgroup A2), are shown in Table 1.

**Table 1:** The comparisons of the clinical characteristics of the study cohorts.

Characteristic		Total *n* = 226	Group A *n* = 113	Group B *n* = 113	*p* (Group A vs. Group B)	Subgroup A1 *n* = 58	Subgroup A2 *n* = 55	Group B *n* = 113	*p* (Subgroup A1 vs. Subgroup A2 vs. Group B)
Age, years [median (Min.–Max.)]		34 (24–44)	35 (24–44)	33 (22–44)	0.256^a^	34 (25–42)^†^	35 (24–44)^†^	33 (22–44)^†^	0.242^d^
BMI, kg/m^2^ [median (Min.–Max.)]		28 (20–38)	28 (20–37)	28 (22–38)	0.102^a^	28.2 (20–37.7)^†^	28 (23.7–35)^†^	28 (22–38)^†^	0.183^d^
Gravidity [median (Min.–Max.)]		2 (1–5)	2 (1–5)	2 (2–5)	0.462^a^	2 (2–5)^†^	2 (1–5)^†^	2 (2–5)^†^	0.387^d^
Parity [median (Min.–Max.)]		1 (1–4)	1 (1–4)	1 (1–4)	0.084^a^	1 (1–4)^†^	1 (1–3)^†^	1 (1–4)^†^	0.206^d^
Time interval since previous CS, months [median (Min.–Max.)]		45.5 (13–132)	46 (13–132)	44 (15–108)	0.978^a^	45 (17–106)^†^	47 (13–132)^†^	44 (15–108)^†^	0.998^d^
Myoma diameter in previous CS, mm [median (Min.–Max.)]		N.A.	50 (20–110)	N.A.	N.A.	20 (20–110)	55 (20–110)	N.A.	0.782
Apgar score [median (Min.–Max.)]	first min	8 (5–9)	8 (5–9)	8 (5–9)	0.551^a^	8 (5–9)^†^	8 (5–9)^†^	8 (5–9)^†^	0.243^d^
	fifth min	9 (7–10)	9 (7–10)	9 (7–10)	0.517^a^	9 (7–10)^†^	9 (7–10)^†^	9 (7–10)^†^	0.798^d^
Neonatal weight, g [median (Min.–Max.)]		3,210 (2,050–4,800)	3,200 (2,050–4,620)	3,260 (2,080–4,800)	0.477^a^	3,205 (2,050–4,520)^†^	3,200 (2,480–4,620)^†^	3,260 (2,080–4,800)^†^	0.584^d^
FGR [*n* (%)]		9 (4.0%)	5 (4.4%)	4 (3.5%)	1.000^b^	2 (3.4%)^†^	3 (5.5%)^†^	4 (3.5%)^†^	0.823^e^
Diabetes mellitus [*n* (%)]		17 (7.5%)	10 (8.8%)	7 (6.2%)	0.449^c^	4 (6.9%)^†^	6 (10.9%)^†^	7 (6.2%)^†^	0.571^e^
Hypertension in pregnancy [*n* (%)]		20 (8.8%)	11 (9.7%)	9 (8.0%)	0.639^c^	6 (10.3%)^†^	5 (9.1%)^†^	9 (8.0%)^†^	0.872^c^
PPROM [*n* (%)]		9 (4.0%)	5 (4.4%)	4 (3.5%)	1.000^b^	2 (3.4%)^†^	3 (5.5%)^†^	4 (3.5%)^†^	0.823^e^
Preterm delivery [*n* (%)]		14 (62%)	7 (6.1%)	7 (6.1%)	1.000^c^	5 (8.6%)^†^	2 (3.6%)^†^	7 (6.1%)^†^	0.554^e^

BMI, body mass index; CS, cesarean; FGR, fetal growth restriction; PPROM, preterm premature rupture of membranes.

Gravidity and parity were calculated before repeated CS.

^a^ Mann–Whitney *U* test.

^b^ Fisher's exact test.

^c^ Pearson *χ*^2^ test.

^d^ Kruskal–Wallis test.

^e^ Fisher–Freeman–Halton test.

^†,‡,§^ The different symbol above the upper character on the “data” indicates that this group is statistically different from the other groups with *p *< 0.05.

All participants had a history of transverse laparotomy in their previous CS. The analysis revealed no statistically significant differences across the groups in terms of median age, BMI, gravidity, parity, and time interval since the previous CS. The median diameter of the fibroid enucleated during previous CS was 20 mm (range, 20–110) in Subgroup A1 and 55 mm (range, 20–110) in Subgroup A2, without statistically significant difference between the subgroups. There was no statistically significant difference between the groups in terms of newborn weight, Apgar scores, premature birth frequency, PPROM, diabetes, and hypertension.

There was an isolated case of complete uterine rupture in Subgroup A1. It occurred in a 34-year-old woman (gravida 3, para 1) who had EM of a 45 mm FIGO type 2 fibroid localized at the posterior wall of the uterus. Her previous CS was 17 months prior to the presentation. She was admitted to the hospital with the chief complaint of abdominal pain at 37 weeks of a previously uneventful pregnancy. The patient was delivered by emergency CS, due to non-reassuring fetal heart rate. On laparotomy, complete uterine rupture at the site of previous LUS incision. A live 3,050 g neonate with an Apgar score of 5/7 was delivered. The patient's preoperative and postoperative hemoglobin levels were 12.6 g/L and 6.5 g/L, respectively, after receiving 1,000 ml of packed red blood cells. The postoperative recovery was unremarkable, and the patient was discharged from the hospital on the second postoperative day.

One patient in Subgroup A1 underwent a repeated CS through a median laparotomy due to complete placenta previa. The patient was a 37-year-old woman (gravida 4, para 1) who had EM of a 100 mm FIGO type 4 fibroid localized at the fundal anterior wall of the uterus. The previous CS was 26 months prior to presentation. The patient was delivered by emergency CS due to excessive vaginal bleeding at the 35th week of pregnancy, the newborn was 2,600 g with an Apgar score of 7/9. Hysterectomy was not required, and 1,000 ml of blood was transfused. No intensive care unit was required for both the mother and baby. The patient was discharged on the third postoperative day. All other patients throughout the study had a transverse relaparotomy.

There was no discernible difference in the length of repeated CS across the groups. The preoperative hemoglobin (Hb) levels in the two main groups were examined, and the results showed no significant differences. In a similar vein, these group's postoperative hemoglobin levels were similar. Subgroup analysis showed that preoperative hemoglobin levels did not differ across the groups. Median postoperative Hb levels also did not reveal significant differences between Subgroups A1 and A2 and Group B [102 (range, 64–127); 104 (range, 79–126); 99 (range, 63–126), respectively] (*p* = 0.051). However, there was an observable trend suggesting a disparity, attributable to the relatively lower Hb levels in Group B. The underlying cause of this trend can be primarily attributed to the disproportionate influence of patients with hemorrhage in Group B. In terms of perioperative transfusion, 6.9% of patients in Subgroup A1, 5.5% of patients in Subgroup A2, and 1.8% of patients in Group B received transfusions, without significant differences between the groups.

The preoperative, intraoperative, and postoperative characteristics of the patients are shown in Table 2.

**Table 2:** The preoperative, intraoperative, and postoperative characteristics of the study cohorts.

Characteristic		Total *n* = 226	Group A *n* = 113	Group B *n* = 113	*p* (Group A vs. Group B)	Subgroup A1 *n* = 58	Subgroup A2 *n* = 55	Group B *n* = 113	*p* (Subgroup A1 vs. Subgroup A2 vs. Group B)
Duration of the repeated CS, min [median (Min.–Max.)]		35 (12–135)	35 (12–135)	36 (17–106)	0.382^b^	35 (18–120)^†^	33 (12–135)^†^	36 (17–106)^†^	0.188^f^
Preoperative Hb, g/L [median (Min.–Max.)]		118 (93–144)	119 (95–144)	117 (93–144)	0.124^b^	118.5 (95–139)^†^	119 (119–144)^†^	117 (93–144)^†^	0.305^f^
Postoperative Hb, g/L [median (Min.–Max.)]		102 (63–127)	102 (64–127)	99 (63–126)	0.080^b^	102 (64–127)^†^	104 (79–126)^†^	99 (63–126)^†^	0.051^f^
Perioperative transfusion [*n* (%)]		9 (4.0%)	7 (6.2%)	2 (1.8%)	0.171^a^	4 (6.9%)^†^	3 (5.5%)^†^	2 (1.8%)^†^	0.233^d^
Myomectomy scar healing [*n* (%)]	good	N.A.	112 (99.1%)	N.A.	N.A.	58 (100%)	54 (98.2%)	N.A.	0.487^e^^,^^!^
	poor	N.A.	1 (0.9%)	N.A.		0	1 (1.8%)	N.A.	
	dehiscence	N.A.	0	N.A.		0	0	N.A.	
Myoma recurrence [*n* (%)]		N.A.	32 (28.3%)	N.A.	N.A.	21 (36%)	11 (20%)	N.A.	0.560^c^^,^^!^
Recurrent myoma diameter^g^, mm [median (Min.–max.)]		N.A.	55 (20–80)	N.A.	N.A.	30 (20–60)	60 (30–80)	N.A.	**<0.001** **^b^ ^,^ ^!^**
Febrile morbidity [*n* (%)]		11 (4.9%)	7 (6.2%)	4 (3.5%)	0.354^c^	4 (6.9%)^†^	3 (5.5%)^†^	4 (3.5%)^†^	0.609^d^
Prolonged hospitalization [*n* (%)]		11 (4.9%)	6 (5.3%)	5 (4.4%)	0.757^c^	2 (3.4%)^†^	4 (7.3%)^†^	5 (4.4%)^†^	0.660^d^

CS, cesarean; Hb, hemoglobin.

^a^ Fisher's exact test.

^b^ Mann–Whitney *U* test.

^c^ Pearson *χ*^2^ test.

^d^ Fisher–Freeman–Halton test.

^e^ Fisher's exact Test.

^f^ Kruskal–Wallis Test.

^g^ The subgroup is formed by cases of recurrent myoma.

^†,‡,§^ The different symbol above the upper character on the “data” indicates that this group is statistically different from the other groups with *p *< 0.05.

^!^ Pairwise *p*-values (Group A1 vs. Group A2).

The value expressed in bold font indicates statistical significance.

The myomectomy scars of all patients in Group A were mostly evaluated as good healing (99.1%), while poor healing was determined in only one patient (0.9%). This poor healing case was a 34-year-old woman with two gravida and one parity (CM). Elective CS was performed at the 39th week of pregnancy, the fetal weight was 3,100 g, and the Apgar score was 8/9. The previous CM was performed 15 months ago. It was determined from the patient records that, in the previous CM, a FIGO type 6 fibroid of 65 mm diameter located in the anterior wall of the uterine corpus had been excised using the serosal myomectomy technique. Preoperative and postoperative Hb levels were 11.9 g/L and 11.1 g/L, respectively. There were no intraoperative or postoperative complications. If we evaluate myomectomy scar healing between subgroups, all cases in Subgroup A1 exhibited good healing outcomes. In Subgroup A2, most of the cases exhibited good healing outcomes (98.2%), while poor healing was determined in only one patient (1.2%). These differences between the subgroups were not statistically significant.

Regarding fibroid recurrence, recurrent myoma locations were different from the previous myomectomy site. The myoma recurrence rate was 28.3% (*n* = 32) in Group A. Within the subgroups, the recurrence rates were 36% (*n* = 21 cases) in Subgroup A1% and 20% (*n* = 11 cases) in Subgroup A2; however, this difference was not statistically significant. Notably, the median diameter of recurrent fibroids in Subgroup A1 was significantly smaller at 30 mm (range, 20–60 mm), compared to Subgroup A2, where the median diameter was 60 mm (range, 30–80 mm) (*p* > 0.001). Febrile morbidity was mostly detected in cases of membrane rupture lasting longer than 12 h or in cases of emergency CS. All patients with febrile morbidity received broad-spectrum antibiotics, and their fever decreased. Regarding febrile morbidity, no discernible variations were seen between the groups. A minimum of 1,000 ml of blood transfusion, severe headaches following a spinal puncture, uterine rupture, midline laparotomy, and extended febrile morbidity were among the patients who required a protracted hospital stay. There were no discernible differences between the groups in terms of extended hospital stays. None of the individuals had any symptoms of ileus or venous thromboembolism.

Surgical complications associated with repeated CS were evaluated according to the Clavien–Dindo classification (16) (Table 3). Most of the patients in the study did not have any of the complications (*n* = 134, 59.3%). Grade I complications were determined in 83 (36.7%) patients. All of these include the additional use of analgesics and antiemetics. No other therapeutic agent was used, and no case of wound infections opened at the bedside was detected. Grade II complications were determined in 9 patients (4.0%), all of whom required blood transfusion. No higher-grade complications were observed. When subgroup analysis was performed, no complications were observed in 32 patients in Subgroup A1 (55.2%), 30 patients in Subgroup A2 (54.5%), and 72 patients in Group B (63.7%). Similarly, Grade I complications were observed in 22 patients in Subgroup A1 (37.9%), 22 patients in Subgroup A2 (40.0%), and 39 patients in Group B (34.5%). Finally, Grade II complications were observed in four patients in Subgroup A1 (6.9%), three patients in Subgroup A2 (5.5%), and two patients in Group B (1.8%). Statistical analysis indicated no significant differences among the groups.

**Table 3:** Evaluation of the long-term postoperative complications (Clavien–Dindo classification) (16).

Grade	Total *n* = 226	Group A *n* = 113	Group B *n* = 113	*p* (Group A vs. Group B)	Subgroup A1 *n* = 58	Subgroup A2 *n* = 55	Group B *n* = 113	*p* (Subgroup A1 vs. Subgroup A2 vs. Group B)
No complication	134 (59.3%)	62 (54.9%)^†^	72 (63.7%)^†^	0.156^a^	32 (55.2%)^†^	30 (54.5%)^†^	72 (63.7%)^†^	0.360^a^
Grade I [*n* (%)]	83 (36.7%)	44 (38.9%)^†^	39 (34.5%)^†^		22 (37.9%)^†^	22 (40.0%)^†^	39 (34.5%)^†^	
Grade II [*n* (%)]	9 (4.0%)	7 (6.2%)^†^	2 (1.8%)^†^		4 (6.9%)^†^	3 (5.5%)^†^	2 (1.8%)^†^	

^a^ Fisher–Freeman–Halton test.

^†,‡,§^ The different symbol above the upper character on the “data” indicates that this group is statistically different from the other groups with *p *< 0.05.

Upon evaluating the intraoperative adhesion values of the patients according to PAI 2013 (17) (Table 4), no adhesions were detected in 70 patients in Group A (61.9%) and 80 patients in Group B (70.8%). Subgroup analysis revealed that 40 patients (69.0%) in Subgroup A1 and 30 patients (54.5%) in Subgroup A2 had no adhesions. Adhesions were determined in 45% of Subgroup A2 and 31.1% of Subgroup A1. The comparison between Group A and Group B revealed no statistically significant difference in the sum of scores per patient (*p* = 0.119). Additionally, within Group A, Subgroups A1 (*n* = 58) and A2 (*n* = 55) showed no significant difference compared to each other as well as Group B in terms of the sum of scores per patient (*p* = 0.074).

**Table 4:** Evaluation of intraoperative adhesions (PAI 2013) (17).

PAI	Total *n* = 226	Group A *n* = 113	Group B *n* = 113	*p* (Group A vs. Group B)	Subgroup A1 *n* = 58	Subgroup A2 *n* = 55	Group B *n* = 113	*p* (Subgroup A1 vs. Subgroup A2 vs. Group B)
Cases without adhesion [*n* (%)]	150 (66.4%)	70 (61.9%)^†^	80 (70.8%)^†^	0.159^a^	40 (69.0%)^†^	30 (54.5%)^†^	80 (70.8%)^†^	0.100^a^
Cases with adhesion [*n* (%)]	76 (33.6%)	43 (38.1%)^†^	33 (29.2%)^†^		18 (31.0%)^†^	25 (45.5%)^†^	33 (29.2%)^†^	
Sum of scores per patient [median (Min.–Max.)]	0 (0–6)	0 (0–6)	0 (0–4)	0.119^b^	0 (0–6)^†^	0 (0–6)^†^	0 (0–4)^†^	0.074^c^

PAI, peritoneal adhesion index.

^a^ Pearson chi-square test.

^b^ Mann–Whitney *U* test.

^c^ Kruskal–Wallis Test.

^†,‡,§^ The different symbol above the upper character on the “data” indicates that this group is statistically different from the other groups with *p *< 0.05.

Logistic regression analysis investigated the relationship between different surgical techniques and long-term postoperative complications, categorized according to the Clavien–Dindo classification (complications were considered based on their presence or absence, without accounting for grades).

### Univariate analysis for whole study groups (*n* = 226)

In the univariate model, comparing Group B (used as the reference group) to Subgroup A2 (OR* *=* *1.463; *p *> 0.05) and Subgroup A1 (OR* *=* *1.427; *p *> 0.05), the results suggest that neither serosal nor endometrial myomectomy significantly increased the risk of long-term complications when added to cesarean section.

### Multivariate analysis for whole study groups (*n* = 226)

Adjusting for age, body mass index, gravidity, parity, and the time interval between the operations, the multivariate model yielded similar odds ratios (1.494 and 1.382 respectively). However, these adjusted odds ratios were not statistically significant (*p *> 0.05), indicating that the additional risk factors adjusted for did not substantially alter the relationship between surgical techniques and postoperative complications.

### Univariate analysis for the myomectomy groups (*n* = 113)

When focusing solely on the myomectomy groups, using Subgroup A2 as the reference, the odds ratio for Subgroup A1 was 0.975 (*p *> 0.05), indicating no significant difference in the risk of postoperative complications between the two myomectomy techniques.

### Multivariate analysis for the myomectomy groups (*n* = 113)

Further adjusting for age, body mass index, gravidity, parity, the time interval between the operations, and the myoma diameter at the first operation, the multivariate model for the myomectomy groups showed an OR of 0.835 (*p* = 0.664) for Subgroup A1 compared to Subgroup A2. This result also did not reach statistical significance, suggesting that the type of myomectomy performed does not significantly impact the likelihood of long-term postoperative complications. These findings underscore that neither serosal nor endometrial myomectomy significantly alters the risk of long-term complications when performed alongside cesarean section, regardless of the patient's age, body mass index, gravidity, parity, or time interval between operations (detailed in Table 5).

**Table 5:** Relationship between surgical techniques and long-term postoperative complications (according to the Clavien–Dindo classification)^a^.

Variable		OR (95% CI)	*p*
Univariate model for whole study groups	Group B (reference)	–	–
	Subgroup A2	1.463 (0.760–2.817)	0.254
	Subgroup A1	1.427 (0.749–2.717)	0.279
Multivariate model for whole study groups^b^	Group B (reference)	–	–
	Subgroup A2	1.494 (0.761–2.932)	0.243
	Subgroup A1	1.382 (0.712–2.683)	0.339
Univariate model for the myomectomy groups	Subgroup A2 (reference)	–	–
	Subgroup A1	0.975 (0.465–2.046)	0.947
Multivariate model for the myomectomy groups^c^	Subgroup A2 (reference)	–	–
	Subgroup A1	0.835 (0.370–1.885)	0.664

OR, odds ratio; CI, confidence interval.

^a^ Logistic regression analysis.

^b^ Adjusted for age, body mass index, gravidity, parity, and time interval between the operations.

^c^ Adjusted for age, body mass index, gravidity, parity, time interval between the operations, and myoma diameter at first operation.

Logistic regression analysis also assessed the relationship between surgical techniques and the incidence of intraoperative adhesions, as classified by PAI 2013 (adhesions were considered based on their presence or absence).

### Univariate analysis for the whole study groups (*n* = 226)

The univariate model indicated that cesarean section combined with serosal myomectomy was associated with significantly higher odds of intraoperative adhesions compared to cesarean section alone (OR = 2.020; 95% CI: 1.036–3.940; *p* = 0.039). In contrast, cesarean section combined with endometrial myomectomy did not show a significant increase in the odds of adhesions (OR = 1.091; 95% CI: 0.548–2.171; *p > 0.05*).

### Multivariate analysis for the whole study groups (*n* = 226)

After adjusting for age, body mass index, gravidity, parity, and the time interval between operations, the odds ratio in Subgroup A2 increased (OR = 2.327; 95% CI: 1.157–4.680; *p* = 0.018), further supporting a significant association with higher adhesion rates. However, the association in Subgroup A1 remained non-significant (OR = 1.062; 95% CI: 0.522–2.158; *p > 0.05*).

### Univariate model for the myomectomy groups

Within the groups undergoing myomectomy, using Subgroup A2 as the reference, Subgroup A1 showed reduced, though not statistically significant, odds of adhesions (OR = 0.540; *p* > 0.05).

### Multivariate model for the myomectomy groups

When further adjustments were made for age, body mass index, gravidity, parity, the time interval between the operations, and the myoma diameter at the first operation, the results for Subgroup A1 indicated lower but not significant odds of adhesions (OR = 0.457; *p* = 0.062). These analyses suggest that serosal myomectomy in conjunction with cesarean section significantly increases the likelihood of intraoperative adhesions, whereas endometrial myomectomy does not significantly alter this risk (detailed in Table 6).

**Table 6:** Relationship between surgical techniques and the existence of intraoperative adhesions (according to PAI 2013)^a^.

Variable		OR (95% CI)	*p*
Univariate model for whole study groups	Group B (reference)	–	–
	Subgroup A2	2.020 (1.036–3.940)	0.039
	Subgroup A1	1.091 (0.548–2.171)	0.804
Multivariate model for whole study groups^b^	Group B (reference)	–	–
	Subgroup A2	2.327 (1.157–4.680)	0.018
	Subgroup A1	1.062 (0.522–2.158)	0.869
Univariate model for the myomectomy groups	Subgroup A2 (reference)	–	–
	Subgroup A1	0.540 (0.250–1.165)	0.116
Multivariate model for the myomectomy groups^c^	Subgroup A2 (reference)	–	–
	Subgroup A1	0.457 (0.201–1.039)	0.062

PAI, peritoneal adhesion index; OR, odds ratio; CI, confidence interval.

^a^ Logistic regression analysis.

^b^ Adjusted for age, body mass index, gravidity, parity, time interval between the operations.

^c^ Adjusted for age, body mass index, gravidity, parity, time interval between the operations, and myoma diameter at first operation.

## Discussion

Since its initial introduction into obstetrics practice, CM is a surgical treatment that is being performed by an increasing number of surgeons. Because of the significant risk of perioperative complications, such as severe bleeding, the need for blood transfusions, the potential for cesarean hysterectomy, and extended hospital stays, CM was avoided in the past (18). Although rare, massive hemorrhage can lead to maternal death (19). It has been stated in many articles that even fibroids <8 cm can be safely removed during cesarean delivery (20), longer recovery times and lower hemoglobin levels are unavoidable (7, 8).

Complications may also arise if fibroids are not removed after a CS because of surgical inexperience or the fibroid's placement. If fibroids are not removed during CS, certain issues could arise. It was formerly believed that fibroids prevented the uterus from involution, which resulted in heavy bleeding during the early puerperal phase (21) or prolapsed from the cervix to the vagina, which led to an infection and myomectomy during this time. Fibroids were suspected of causing infertility, miscarriage or preterm birth of the fetus, excessive monthly flow, and symptoms connected to surrounding organ pressure, late after the delivery. Pregnant women who experience severe pain that is unresponsive to medical intervention may be more inclined to choose CM (22).

During CS, eliminating fibroids has several benefits. Due to increased myometrial elasticity during pregnancy, it will be easier to distinguish healthy and fibroid myometrial tissue, and myoma removal becomes easier. During pregnancy, uterine expansion is more noticeable than fibroid growth. This reduces tissue damage when surgeons remove fibroids from smaller incisions. Significant contractions following CS and uterine involution reduce the quantity of bleeding from the fibroid bed. It saves the patient from myoma symptoms and possible subsequent myomectomy. The serosal technique has been used for a very long time to accomplish myomectomy during CS. SM can remove easily accessible fibroids that are near the serosal surface (G7, G6, G5, G4, G3, G2-5 fibroids). By using an endometrial approach, fibroids that are deeply ingrained (G4, G5, G6 fibroids) in the myometrium and near the endometrium (G1, G2, G3, G2-5, fibroids) can be removed. The application of hysteroscopic myomectomy served as the model for EM. The EM can also be performed for fibroids located in the posterior uterine wall or fibroids located in isthmic or cornual regions that cannot be removed by SM (23). Moreover, the EM can be combined with SM for the removal of all fibroids present during CS, and those fibroids located on the incision line can be removed by the trans-incisional route.

There are no internationally established guidelines for my myoma management during the CS. Most of the classical textbooks and internationally established opinions advise against performing myomectomy during CS except in very few situations (24). On the other hand, literature data provide a lot of publications promoting CM ( 6, 11, 12, 20, 23, 25). Moreover, five available meta-analyses failed to document any major complications of CM and documented its safety and feasibility in properly selected cases (8, 9, 10, 22, 26).

Although short-term outcomes were reported in many publications, studies and meta-analyses of long-term outcomes of CM are lacking (9, 10, 26). In their recent review, Sparic et al. evaluated six studies comparing the short-term results of CM according to the myomectomy technique. This study, which once again demonstrates the reliability of CM, states that none of the techniques is superior to the other and well-designed studies are needed for long-term outcomes (27). Akkurt et al. (12) evaluated 32 out of 91 women who had CM for their pregnancies after CM for preterm delivery, uterine rupture, and placental adhesion abnormalities, detecting a very low maternal morbidity rate for those factors (9.3%). Adesiyun et al. (28) reported that CM has no negative impact on the obstetrical outcomes of 29 women who had previous CM. Yıldırım Karaca et al. (29) compared obstetrical and neonatal outcomes (Apgar score, neonatal birthweights, birth weeks) after EM and SM in two groups, reporting no statistically significant difference in both groups.

In our study, we evaluated long-term obstetrical outcomes of women who had previous CM and for neonatal Apgar scores, birthweights of babies, fetal growth restrictions, diabetes mellitus, PPROM, and preterm delivery, and our results showed no significant difference in both methods.

We found a uterine rupture in one of the cases assigned to Subgroup A1; however, the ruptured site was from the lower uterine segment (LUS) and not the prior CM site, demonstrating that the rupture and CM are unrelated. The incidence of placenta previa in the general population ranges from 0.28% to 1.5% (30). In our 113 cases, we only recorded one case with placenta previa. However, Akkurt et al. and Adesiyun et al. reported higher rates of placenta previa, understandably due to a very low number of studied populations (12, 28). Contrary to their report, Huang et al. performed EM to 63 cases and then performed CS to the same 63 cases, and the rate of placenta previa was determined to be the same in both groups. The author concluded that EM does not cause an increase in the risk of placenta previa (31).

The length of CS, preoperative and postoperative hemoglobin levels, the need for blood transfusions, febrile morbidity, and extended hospital stays were among the perioperative data that we did not observe to differ significantly from one another during our analysis. It is possible to identify no discernible change because both groups had CS.

According to the study of Fauconnier et al. (32), the rate of fibroid recurrence following abdominal myomectomy ranges from 15.4% to 62% if transvaginal ultrasound examination is the recommended approach to assess it. Kotani et al. followed women with previous open myomectomy for fibroid recurrence and found 5.3%, 34.2%, 46.9%, and 63.4% in their 1, 3, 5, and 8 years of follow-up, respectively (33). From the publication, the mean recurrence rate was expected 40% during follow-up. Our study revealed a 28% recurrence rate, and the mean time for second CS was 3.9 years. The low recurrence rate in our study may be explained by the removal of palpable and visualized fibroids during CS instead of transvaginal ultrasound examination. The disadvantage of EM is that we cannot determine small fibroids. Literature is lacking about the long-term outcomes of healing of CM scar. Cobellis et al. published two consecutive articles in the same year. In their first article, they compared ultrasonographic findings of myomectomy scars in both the CM and myomectomy groups, while in the second publication, they compared myomectomy scar healing of previously evaluated patients during CS (34, 35). Both studies showed that scar healing is much better in the CM group. Moreover, Adesiyun et al. (28) evaluated 29 women with previous CM, with 13 women who delivered by vaginal route and 16 women who had CS. In both groups, none of the cases had uterine dehiscence or uterine rupture. In our investigation, we detected a good healing rate in 99.1% of patients, where we evaluated the scar healing by palpation during CS. Our findings are matched with the limited data present in the literature.

Myomectomy scar pregnancy is one of the rarest myomectomy side effects. Zhu et al. (36) published their case along with a review study that featured seven myomectomy scar pregnancies. Out of the eight myomectomy scar pregnancies, three cases were noted following laparoscopic myomectomy, and five were the consequence of abdominal myomectomies. Although it is theoretically feasible, none of the few documented cases of myomectomy scar pregnancy were caused by CM (37, 38).

An inevitable complication after all abdominal surgeries is adhesion formation. The adhesion rates after abdominal myomectomy range from 28.1% to 81% (39, 40). Adhesion formation after CS was 24%–46%, 43%–75%, and 83% in second, third, and fourth CS, respectively (41, 42).

Turgal et al. (13) stated the adhesion rate in CS after serosal myolysis and SM performed during previous CS as 19% and 41%, respectively. When these rates were compared with the CS after CS group (21.1% adhesions), no statistically significant difference was detected. This finding was further supported by Akkurt et al., where they reported a 25% adhesion rate during CS in 32 women who had previous SM (12).

In our investigation, Group B had a rate of adhesion development of 29.2% compared to Group A's 38.1%; nevertheless, this difference was not statistically significant. Mercorio et al. (43) in their recent review evaluated adhesion formations after myomectomy and stated that the size of the uterine serosal incision is related to adhesion. Based on this result, since the uterine serosa is not damaged in the EM technique, less adhesion is expected compared to the SM technique. Moreover, women with prior SM during CS were observed to have higher adhesion development in their second CS as opposed to women with EM during CS, according to Yıldırım Karaca et al. (29). We realized that Subgroup A1 had a 31% adhesion rate and Subgroup A2 had a 45.5% adhesion rate in the multiple group comparisons. Although Subgroup A2 had a higher rate, this difference was not statistically significant. However, further evaluation through logistic regression analysis to assess the risk of adhesions across different surgical groups revealed a different pattern. The analysis showed that the likelihood of intraoperative adhesions was significantly higher between Subgroup A2 and Group B (OR = 2.020; 95% CI: 1.036–3.940; *p* = 0.039). This association was found to strengthen further when adjusted for demographic and preoperative variables (OR = 2.327; 95% CI: 1.157–4.680; *p* = 0.018). Conversely, there was no significant increase in the risk of adhesions between Subgroup A1 and Group B (Table 5). The results obtained in this evaluation seem to be consistent with the literature. To draw any conclusions on adhesion formation following myomectomy, a more extensive series should be examined in future research.

The growing incidence of CS globally raises awareness of the risks associated with the procedure, with heavy bleeding during CS being the most common consequence (7%) (44). The range of intestinal blockage is 0.05%–0.2% (45). There were incredibly few other organ injuries. Delporte et al. (46) examined 882 women who had CS, 17% of whom had repeated CS, and assessed the problems associated with the procedure using the Clavien–Dindo classification. In accordance with this classification scheme, problems were noted in Grades I, II, and III at 89.2%, 8.7%, and 2.0%, respectively. We added severe postoperative pain, analgesic use, and antiemetic use due to severe nausea and vomiting to Grade I and reported a 36.7% Grade I complication rate using the same classification approach. In 4% of the instances, we found bleeding and the need for blood transfusions because of the bleeding, classifying them as Grade II.

We did not uncover any Grade III complications in our investigation. While the Grade I complication rate was lower than the literature, the Grade II complication rate was comparable. Regarding surgical complications, there does not appear to be a statistically significant difference between the groups and subgroups. Further investigation into the relationship between surgical techniques and long-term postoperative complications across different groups showed no significant differences. The results from both univariate and multivariate analyses indicated non-significant odds ratios among Group A1, Group A2, and Group B, demonstrating that different myomectomy procedures together with CS do not substantially alter the risk of long-term complications comparing CS alone. Adjustments for factors such as age, body mass index, and time intervals between operations did not change these findings (Table 6).

This study's first strength was its ability to consistently disclose both short- and long-term obstetric and surgical outcomes through comparative analysis of groups similar in terms of case numbers and fundamental features. The sample size was adequate for a thorough analysis of the primary and secondary outcomes, which is the study's second strength. There are various restrictions on this study. We conducted a multicentric, retrospective investigation. Furthermore, standardizing and avoiding bias in the gathering of data on adhesion and scar healing is a challenging task. The standardization of the data is limited by the various follow-up periods (1–10 years) when evaluating the long-term outcomes of our investigation. Data comparing the long-term effects of SM and CM are scarce. There are not many reports and few cases in the literature yet that can be compared to the findings of our investigation.

## Conclusion

This study demonstrated that obstetrical and perinatal outcomes were unaltered in pregnancies that followed CM. Surgical problems from CS after CM are similar to those from multiple cesarean sections. This applies to both categories of CM techniques. Repetitive CS and CM are comparable in terms of how long-term adhesions are created. However, because there is no serosal incision in EM, there is less adhesion formation than in SM. Both the trans-endometrial and serosal techniques of CM are safe, effective, and produce long-lasting outcomes, thus obstetricians can safely utilize them.

## Data Availability

The raw data supporting the conclusions of this article will be made available by the authors, without undue reservation.
